# Preparing Superconducting YBCO Colloids via a Top-Down Processing Route for Applications in Soft Composites

**DOI:** 10.1021/acsomega.5c08369

**Published:** 2025-11-12

**Authors:** Harrison Reinheimer, Mathew M. Maye

**Affiliations:** Department of Chemistry, 2029 Syracuse University, Syracuse, New York 13244, United States

## Abstract

High-temperature superconductors (HTSCs) are critical materials for magnetic, power, and energy storage applications. The cuprate-based HTSCs, such as yttrium barium copper oxide, YBa_2_Cu_3_O_7–*δ*_ (YBCO), allow for superconducting devices at a critical temperature (*T*_c_) above 90 K, which is easily accomplished via cryogenic liquid nitrogen. One challenge of HTSCs is the need for high crystallinity of an oxygen-deficient perovskite solid, which can only be achieved via high-temperature solid-state synthesis and subsequent annealing. HTSCs have ceramic-like physical properties and are not easily processed or chemically synthesized to colloidal sizes at scale. Herein, we developed a multistep top-down processing method to break YBCO into finer ligand-stabilized colloids, which could then be suspended in solvents as a concentrated paste or ink. We show the potential of such inks by dispersing YBCO in poly­(dimethylsiloxane) (PDMS) elastomer molds, creating superconducting soft composites. Using this approach, we found that chemical stoichiometry and crystal structure of the parent YBCO are preserved, and thus the final colloids are also superconductive. Powder X-ray diffraction and scanning electron microscopy confirmed the preservation of crystal structure and grain morphology, while Meissner effects and quantum locking observations, coupled with magnetic susceptibility measurements, revealed trends in superconductivity and *T*_c_. Finally, we show how the soft YBCO–PDMS elastomers have superconductive properties that are a function of the YBCO weight percent, which can be easily tailored.

## Graphical abstract

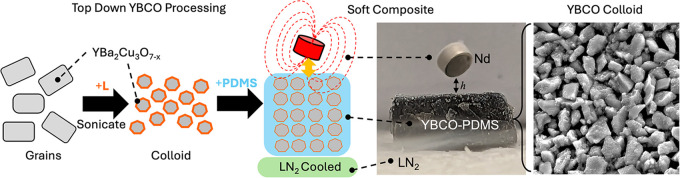

## Introduction

Yttrium barium copper oxide (YBa_2_Cu_3_O_7−δ_, YBCO) and other rare-earth copper oxides are important high-temperature superconductor (HTSC) materials that exhibit superconductivity at critical temperatures (*T*_c_) of >90 K.^1–3^ Using HTSCs allows for the efficient use of high electrical currents to create magnetic fields that are otherwise difficult and thus allows for improvements in technologies ranging from medical imaging and scientific research to low-loss power transmission lines^4,5^ and quantum devices.^6^ The material itself is hard and brittle owing to its ceramic nature, and the superconducting state often requires a continuous single-crystal-like microstructure for the best electrical and magnetic properties. To date, HTSCs are best prepared using solid-state synthesis using compressed metal oxide precursors annealed at high temperatures, often for long hours at temperatures exceeding 1000 °C, and further calcinated for similar times and conditions to create the required oxygen deficiencies and nonstoichiometry.^7,8^ These routes are straightforward and highly reproducible at scale. However, from the perspective of chemists or materials scientists, the lack of other synthetic or processing routes to create HTSCs limits applications to only those that use the macroscale material.^9^ There thus remains a need to create or easily process HTSCs into thin films, lithographic patterns, colloids, or even nanostructures in order to harness superconductivity for synergistic applications in self-assembly, energy transfer, near field effects, heterostructures, or quantum devices. Wet chemistry has been used previously to create HTSC materials with modest performance, typically via processing metal halide salts via microwave irradiation,^10,11^ electrospinning,^12^ sol–gels,^13–16^ micelles,^16^ solution precipitation,^17,18^ or chemical/physical deposition.^19–21^ However, in these approaches, the first synthetic steps, mixing of metal salts, are challenged by differences in solubility of a precursor as well as the need to remove by-products, like chlorides, water, and carbonates. In addition, these products still require requisite calcination, resulting in an uncontrolled final morphology or film.^22^ Researchers have also used milling,^1–3,23–25^ ultrasonication,^26–28^ etching,^29^ and heating of inkjet-printed films.^30^

One novel alternative approach has been to dissolve a functional YBCO substrate into soluble precursors, such as with trifluoroacetate (TFA), and to use those Y-, Ba-, and Cu-TFA products for chemical solution deposition (CSD) to prepare epitaxial thin films.^31^ This route is effective due to the predetermined metal stoichiometry of the TFA solution as well as the single TFA by-product, whose required decomposition or calcination conditions have been determined. In addition to using this approach for epitaxial YBCO thin films, researchers have also used it to create secondary phases,^32^ as well as to integrate nanoscale materials, like HfO_2_ ^33^ and ZrO_2_ ^34,35^ in YBCO, and YBCO formed in the presence of perovskites,^36^ to influence crystal growth and vortex pinning centers.^37^ Another approach to dissolve YBCO into soluble precursors for CSD thin films is to use propionic acid in the presence of TFA or trifluoroacetic anhydride (TFAA)^38^ or to form metal propionates in fluorine-free conditions.^39,40^ Moreover, recent reviews have highlighted the need for HTSC advances using soft matter approaches^41^ for quantum devices and power applications.^42^ Recently, there have been several novel studies to create low-temperature metal superconductors using nanoscale templates, such as the deposition of niobium within DNA origami.^43^ In addition, lead nanoparticles have been shown to have superconductivity and quantum locking at low temperatures.^44^ Despite these studies, there still is a large knowledge gap as it relates to creating HTSCs with meso- to nanosizes that can integrate into many modern nanotechnology, lithography, or soft-lithography approaches.

In this study, we prepared model YBCO substrates and then used mechanical, ultrasonic, and chemical means to break them into smaller grains that were easily handled as an ink or paste. The role that ligand functionality and concentration had on etching and grain sizes was studied. Powder X-ray diffraction showed that the crystal structure was maintained through this processing route, and scanning electron microscopy confirmed the colloidal grain sizes and distributions. Importantly, the superconducting nature of the YBCO colloids was preserved, eliminating the need for an additional calcination step. This was observed visually via quantum locking experiments and molecularly using magnetic susceptibility measurements. Finally, a proof of principle demonstrating the versatility of YBCO colloids was performed by incorporating them into poly­(dimethylsiloxane) (PDMS) elastomers, which could be patterned and shaped and had weight-percent-determined magnetic properties.

## Experimental Section

### Materials

Barium carbonate (BaCO_3_, 99.8%) was purchased from Alfa Aesar. Acetone ((CH_3_)_2_CO, 99.5%), chloroform (CHCl_3_, >99.8%), copper­(II) oxide (CuO, >99.0%), octadecene (ODE, 90%), oleic acid (OAc, 90%), oleyl amine (OAm, 90%), trioctylphosphine (TOP, 90%), toluene (Tl, >99.5%), methanol (MeOH, >99.8%), and yttrium­(III) oxide (Y_2_O_3_, 99.99%) were purchased from Sigma-Aldrich. Trioctylphosphine oxide (TOPO, 90%) was purchased from Strem Chemicals. Acetonitrile (CH_3_CN, 99%) and ethanol (EtOH, 99%) were purchased from VWR Chemicals. Poly­(dimethylsulfoxane) (PDMS) base and activator (Sylguard 182 Silicone Elastomer Kit) were purchased from Dow Chemicals. Aluminum-coated neodymium rare-earth disk magnets (Nd) were purchased from K&J Magnetics Inc.

### Solid-State Synthesis

Solid YBa_2_Cu_3_O_7‑δ_ (YBCO) substrates were synthesized via solid-state synthesis.^45^ First, in a mortar and pestle, 1 mmol Y_2_O_3_, 8 mmol BaCO_3_, and 12 mmol CuO were ground together to form a uniform mixture and then pressed into a cylindrical pellet using a die (13 mm, International Crystal) and hydraulically pressed at 8 tons for 30 min in air. Next, the solid pellet was collected and calcined in air in a high-temperature furnace at 930 °C for 10 h at a heating rate of 5 °C/min and then cooled at the same rate to room temperature. After heating, the pellet was obtained as a hard, continuous black substrate with ceramic-like mechanical properties and used as is.

### YBCO Processing

The YBCO substrate made above was then broken into pieces and ground into a fine powder using a mortar and pestle without additional mechanical sifting. Next, ∼1 g of YBCO powder was added to 5 mL of ODE in a 10 mL crimp-top serum vial, and mixtures of OAm/OAc or TOP/TOPO were added at different molar ratios (see Table 1). These mixtures were degassed with Ar or N_2_ gas and then heated for 6 h at 65 °C using an ultrasonic bath (120 W, 45 kHz) that had a total reservoir of 800 mL of water. The water level was constantly monitored and adjusted as needed. After sonication, the samples were centrifuged at 10,000 rpm and the ligand supernatant was decanted. The powder was then redispersed in CH_3_CN/CHCl_3_ (1:1) or EtOH and centrifuged. After multiple repeated purification steps, the final YBCO was dried under Ar or N_2_ and then redispersed in a solvent, like Tl or ODE.

**Table 1:** Molar Ratios Used in the Preparation of Colloidal YBCO

	YBCO:ligand molar ratios			
system	[YBCO]	[OAc]	[OAm]	[TOP/TOPO]
S	1			
A1	1	8	8	
A2	1	8	15	
A3	1	32	15	
T1	1			45
T2	1	30		22
T3	1	75		22

### PDMS–YBCO Composites

A Sylguard 182 silicone elastomer kit was used to prepare PDMS molds containing controlled weight percentages (wt %) of YBCO. First, a PDMS negative mold was created using a base-to-activator ratio of 5:1 with 5–10 rectangular aluminum spacers within, which was cured at 140 °C for 12 h, cooled to room temperature, and the aluminum spacers were removed. Next, the PDMS–YBCO composites were prepared using a 10:1 base:activator ratio with the YBCO samples added at the desired weight. Before being added to the PDMS negative mold created above, the surface was rinsed with a commercial detergent, then rinsed with EtOH, and dried. The PDMS–YBCO was then cured as described above, removed from the mold, and used as is.

### Instrumentation

Powder X-ray diffraction (XRD) was performed on a Bruker D2 phaser diffractometer using Cu Kα radiation (λ = 1.5406 Å) with sample powders dried on a zero diffraction plate XRD holder (MTI Crystal Inc.). Diffraction patterns were compared to reference patterns provided by the Crystallography Open Database (COD) and fit via DIFFRAC.EVA software (Bruker Inc.). Scanning electron microscopy (SEM) micrographs were collected on a JEOL JSM-IT100 microscope with sample powders dispersed onto carbon tape (Ted Pella). Before imaging, the samples were coated with a thin layer of gold via sputtering. The ultraviolet–visible (UV–vis) spectroscopy measurements were collected on a Varian Cary 100 Bio UV–vis spectrophotometer with samples dispersed in a solvent. Thermogravimetric analysis (TGA) was done on Perkin-Elmer Pyris 1 using a N_2_ purge and dried powders. Fourier transform infrared (FTIR) spectroscopy was performed on a Thermo Scientific Nicolet 6700 spectrometer with an LN_2_-cooled MCT detector and sample powder dispersed on a diamond ATR crystal. Inductively coupled plasma optical emission spectroscopy (ICP-OES) was performed on a Perkin-Elmer Avio 220 ICP-OES system using samples digested in 5% nitric acid and then diluted in ultrapure water. Magnetic susceptibility measurements of weighed sample powders were performed using a vibrating sample magnetometer (VSM) on a Quantum Design DynaCool Physical Property Measurement System (PPMS) instrument at the Cornell Center for Materials Research (CCMR).

## Results and Discussion

Scheme 1 shows the general hypothesis and strategy adopted for the top-down preparation of YBa_2_Cu_3_O_7−δ_ colloids, denoted for simplicity as YBCO. Briefly, a bulk YBCO sample was first prepared by solid-state synthesis and further calcination and denoted as “substrate” for simplicity (a). This substrate was then mechanically broken into polycrystalline grains (b) and then chemically (c) broken down with ligands (L) into smaller colloidal forms (c) with the aid of sonication. These YBCO grains and colloids could either be used as is or suspended in a solvent to form a paste or ink, which was then readily mixed with an elastomer and cured into a PDMS soft composite (d) with different shapes at controlled weight percentages for numerous applications and devices.

**Scheme 1:**
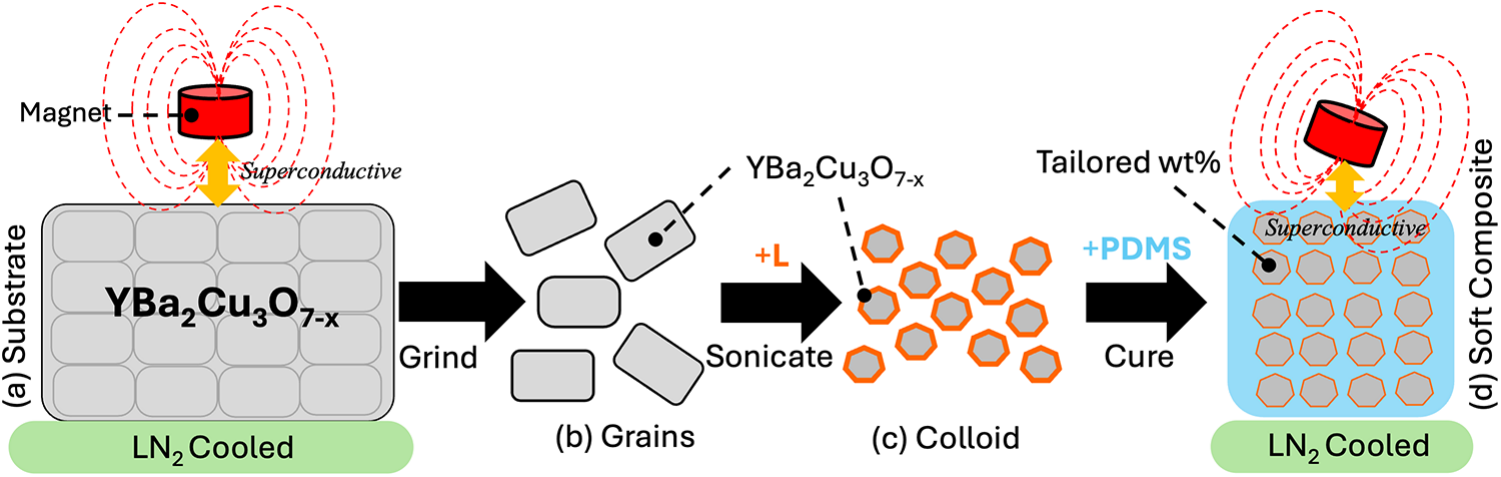
Idealized Schematic of the Top-Down Processing Approach to Create Colloidal YBCO^a^

a A bulk YBCO substrate was first prepared (a) and then ground to fine grains (b), which were then chemically etched during sonication with ligands into colloidal YBCO (c). Finally, the colloidal YBCO was easily processed with PDMS, forming a YBCO–PDMS soft elastomer (d). The superconducting properties of the YBCO were preserved at each step (shown).

The YBCO substrate was first synthesized in-house via solid-state methods.^45^ Briefly, 1:2:3 molar ratios of yttrium oxide, barium carbonate, and copper­(II) oxide were ground together before being pressed into a gray pellet, which was then calcined in air at 950 °C for 10 h. Upon annealing, the YBCO substrate became black, hard, and brittle owing to its ceramic nature. Figure 1a shows the characterization of the substrate by powder X-ray diffraction (XRD) (i) and scanning electron microscopy (SEM) (ii). The XRD reveals an orthorhombic, oxygen-deficient perovskite structure that closely resembles that of a YBa_2_Cu_3_O_7−δ_ standard, as shown by the reference indices and unit cell. SEM revealed the microstructure of fused uniform grains that were smooth and connected in a network, which showed some porosity at the pellet surface. The photograph in Figure 1a­(ii) shows the YBCO pellet when cooled by LN_2_ and suspending a Neodymium (Nd) permanent magnet, showing the Meissner effect, indicating superconductivity property below its 90 K *T*_c_. Superconductivity below the critical temperature (*T*_c_) was also confirmed via magnetic susceptibility measurements,^46^ as described below.

**Figure 1:**
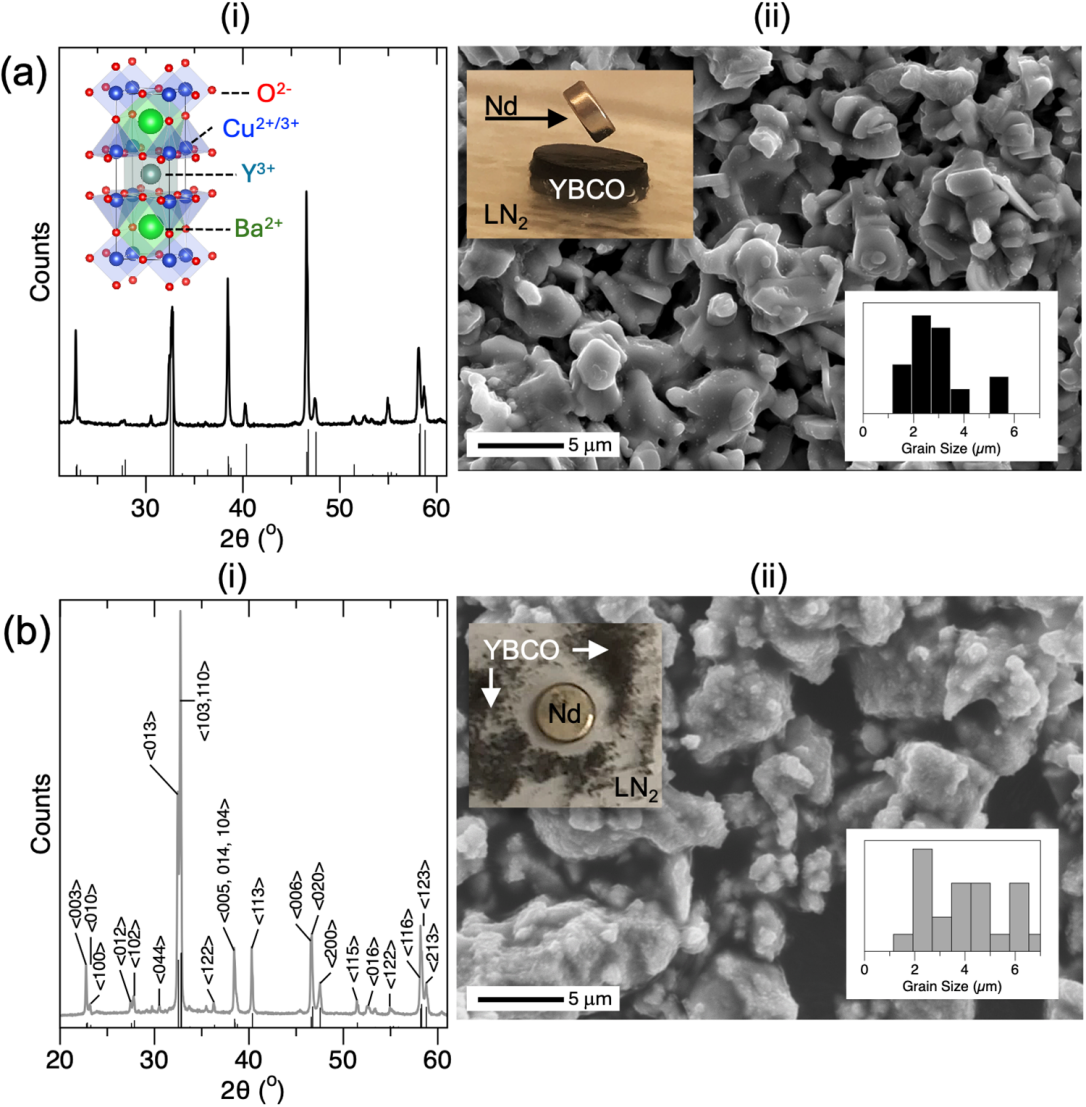
(a) Characterization of the synthesized YBCO pellet by XRD (i) and SEM (ii). The inset shows the YBa_2_Cu_3_O_6.8_ unit cell, the photograph of an LN_2_-cooled YBCO pellet and Nd magnet, and the size analysis histogram of SEM. (b) Same set of results for the ground YBCO substrate. The insets show the photograph of LN_2_-cooled powders repelled by an Nd magnet and the size analysis histogram of SEM. Reference YBa_2_Cu_3_O_6.8_ diffraction and Miller indices from the COD-1008351 reference.

Figure 1b shows a similar set of XRD (i) and SEM (ii) results for the ground YBCO substrate. The XRD (i) showed the same cuprate crystal structure (i) but with an intensity pattern that more closely resembled the reference. Unlike the YBCO substrate, which showed an intensity pattern that suggested a layered substructure, the powder had a pronounced diffraction at 2θ = 32.7°, suggesting some degree of preferred etching. The SEM showed rougher and clearly disconnected grains when compared with the pellet, and grain sizes were found to be ∼4.4 ± 2.0 μm, which was slightly larger than the pellet grains (Figure 1a­(ii) ∼3.0 ± 1.1 μm), which we attribute to the SEM imaging only the surface grains of the pellet, see histograms. The molar ratio of the metals in YBCO was confirmed via ICP-OES (Table S1).

To chemically process the ground substrate, six systems were studied where YBCO was suspended in the noncoordinating solvent octadecene (ODE) and then combined with ligand mixtures that were sonicated for 6 h at 65 °C, before being collected via centrifugation and purified with a nonsolvent. Table 1 shows the systems and their ligand compositions. In series A, oleic acid (OAc) and oleyl amine (OAm), at different molar ratios, were used. In series T, trioctylphosphine (TOP) and trioctylphosphine oxide (TOPO) were added alone (T1) or in combination with OAc (T2–T3). After the reactions, the suspended YBCO was purified free of excess ligands and solvent via multiple cycles of centrifugation and solvent. The final products were dried under a stream of N_2_ gas and resuspended in toluene.

For example, Figure 2a shows a photograph of the A1 YBCO product suspension, and photographs of the other products are shown in Figure S1. These samples were then analyzed and compared. Figure 2a shows the XRD (i) and SEM (ii) results for YBCO products from system A1, which had a ligand molar ratio of [YBCO]:[OAc]:[OAm] = 1:8:8. The diffraction patterns closely resemble those of the YBCO powder above and match the YBa_2_Cu_3_O_6.8_ reference pattern shown. The SEM shows irregular grain shapes, and size analysis determined an approximate grain size of ∼3.1 ± 0.9 μm, as shown by the histogram.

**Figure 2:**
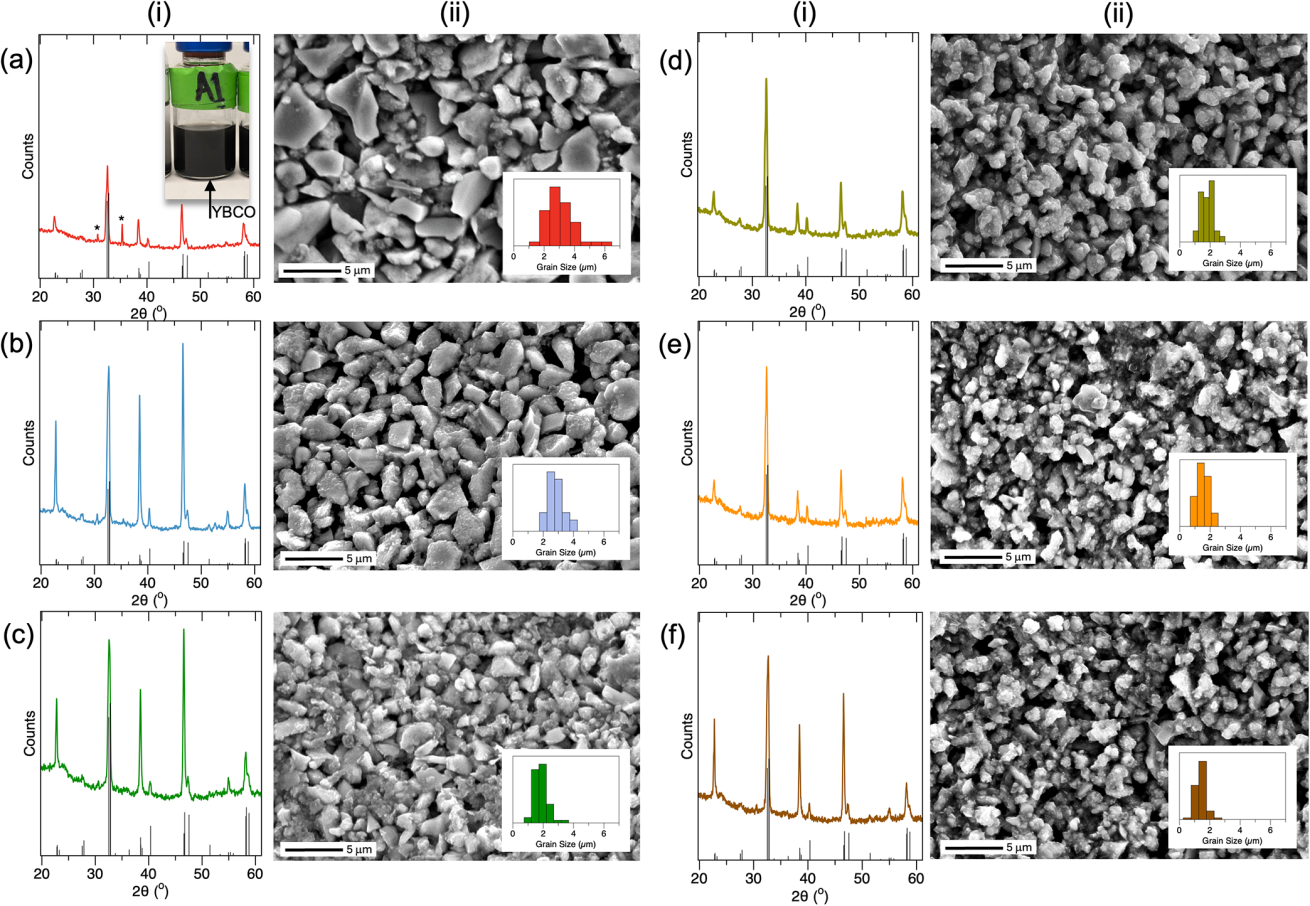
Representative analysis of samples A1 (a), A2 (b), A3 (c), T1 (d), T2 (e), and T3 (f) using XRD (i) and SEM (ii). The insets show the photograph of an A1 colloid suspension (a-i) and grain size analysis histograms for each SEM. Additional SEM magnifications are shown in Figures S2 and S3 for additional SEM. Reference YBa_2_Cu_3_O_6.8_ diffraction from COD-1008351.

Figure 2b,c shows a similar set of XRD (i) and SEM (ii) data sets for the A2 and A3 systems. Two differences emerged when using these ligand concentrations. First, while the diffraction still aligns with the reference, the systems had a different intensity pattern, with pronounced intensities at 2θ = 22.7° and 38.5°, suggesting further preferred orientation of the etching,^47–49^ which is potentially aided by the sonication treatment, as cavitation is more likely to occur at crystal phase boundaries and defects within the material.^50,51^ No significant diffraction broadening was observed, and full width at half-maxima (fwhm) was ∼0.5° at 2θ = 32.5°, for instance. The SEMs show a similar shape of the YBCO, but with a measurable decrease in grain size to ∼2.8 ± 0.5 and ∼1.8 ± 0.4 μm, respectively. Additional SEM micrographs at different magnifications are shown in Figures S2 and S3. As shown in Table 1, sample A3 had the highest overall ligand concentration and the highest [ligand]:[YBCO] ratio. It is important to note that additional studies at higher ligand concentrations, >50× for instance, resulted in complete dissolution of the YBCO substrate, and only blue soluble solutions were obtained.

The combination of OAm with OAc is known to form protonated OAm^+^ and deprotonated OAc^–^ pairs in solution. This combination of ligands is standard in the quantum dot (Qdot) synthesis fields, and a number of studies have shown how they encapsulate the inorganic Qdot or assist in nucleation and growth.^52,53^ In this system and at the concentrations employed, etching of cations will be promoted by the OAc, while the OAm can coordinate to CuO polyhedra.

We next studied a TOP/TOPO system (T1), and two TOP/TOPO plus OAc systems (T2–T3), as shown in Table 1. The TOP/TOPO system was chosen because it has been shown to bind strongly to the interface of Qdots and other ionic nanostructures, as well as because of its differences in physical and chemical properties.^54^ For example, compared to OAm and OAc, TOP/TOPO has a higher surface tension, which has been shown to significantly enhance the fracturing energy (i.e., cavitation) during sonication.^55–58^ During cavitation, lower-surface-tension solvents result in lower cavitation energies, and bubble nucleation occurs at the surface of interfaces, while high-surface-tension solvents enable bubble collapse with a greater shear force.^50,59^ However, it is important to note that both ligand systems were also dissolved in the ODE solvent, which itself has a surface tension of ∼24 nM/m. Chemically, TOP is a softer Lewis base compared to OAm or OAc, or their likely deprotonated (OAc^–^) and protonated (OAm^+^) forms, but it can also coordinate strongly to the oxygens in the CuO_6_ or CuO_4_ polyhedra.^60^ TOPO is a harder Lewis base, likely coordinating strongest to Cu^2+/3+^ cations, followed by the Y^+^ and Ba^2+^ cations. This coordination and dissolution likely occur first at the interface of the larger grains and the interconnecting regions between grains.

Figure 2 also shows the characterization results of T1 (d), T2 (e), and T2 (f) systems using XRD (i) and SEM (ii). Interestingly, the XRD results show that the diffraction at 2Θ ≈ 38° planes is lower in intensity for T1 (d) and T2 (e) but reappears for T3 (f). Moreover, the SEM (ii) showed more monodispersed and smaller grain sizes of ∼2.2 ± 1.1 μm (d-ii), ∼1.6 ± 0.4 μm (e-ii), and ∼1.3 ± 0.3 μm (f-ii) for T1, T2, and T3. Isolated YBCO could also be observed, as shown in Figure S4, by using SEM and transmission electron microscopy (TEM), which shows some populations of YBCO with sizes of 100–300 nm.

The A- and T-series products had similar optical and surface chemistry properties. Neither exhibited any specific absorption in the ultraviolet–visible spectrum (UV–vis), which is shown in Figure S5a, where only scattering/turbidity is observed, which is not surprising considering the sizes given above. The OAm/OAc or TOP/TOPO modification of the surface of the purified YBCO was observed by Fourier transform infrared spectroscopy (FTIR), as shown in Figure S5b, where a strong ∼CH_2_ signal is detected. The organic ligand mass of the YBCO was determined to be 10–14% of total surface mass by thermogravimetric analysis (TGA), as shown in Figure S5c. Considering the size of the colloid, this mass loss is higher than one might expect, which suggests that even after purification, some free ligands remain, possibly due to bilayer formations.

As described above, the YBCO powder and A- and T-series YBCO colloids showed qualitative superconducting characteristics when cooled via LN_2_. The Meissner effect could be observed, and the samples exhibited strong quantum locking. To quantitatively measure this, the samples above were measured by a magnetic susceptibility measurement vibrating sample magnetometer (VSM) Physical Property Measurement System (PPMS) instrument. Interestingly, in contrast to magnetic materials, the magnetic susceptibility of HTSCs shows a repulsion of the applied magnetic fields below *T*_c_, which shows as a negative magnetic moment in a hysteresis plot.^61^

Figure 3 shows a set of susceptibility measurements for the ground YBCO substrate and products from the A1–A3 systems at temperatures of 100 (a), 70 (b), and 5 K (c). At 100 K, only weak paramagnetic moments are observed, and when the magnetic moment is normalized as emu/g, the colloids were more magnetic than the YBCO substrate (black lines), likely due to the presence of defects at the surface of the colloids and unsaturated metal cation environments.

**Figure 3:**
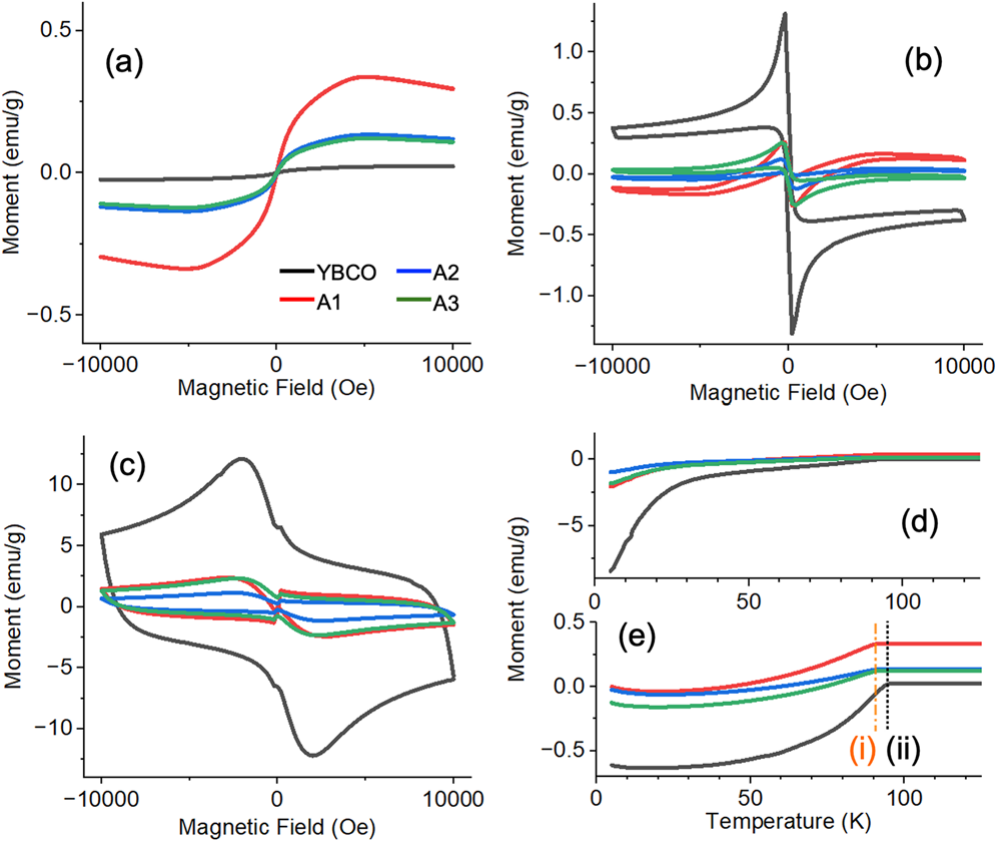
Magnetic susceptibility measurements at 100 (a), 70 (b), and 5 K (c) for YBCO ground substrate (black) and samples A1 (red), A2 (blue), and A3 (green). (d) Zero-field-cooled (ZFC) measurements. (e) Field-cooled (FC) measurements, with observed *T*_c_ A1 (i) and YBCO (ii) highlighted. The same color legend is given in each graph.

At 70 K, the sample is below the *T*_c_ of ∼93 K for YBCO, and the negative moments indicating superconductivity are observed. There is a maximum in the moment at only an applied field of only a few hundred Oe, and then it decreases at higher fields, which indicates the field lines can penetrate the material (or grains), which is a characteristic of the Type II superconducting behavior of YBCO. At the much lower temperature of 5 K (c), there is a pronounced hysteresis, due to the YBCO more effectively repelling the field. The transition to the superconductive state could be observed by field-cooled (FC, Figure 3e) measurements, which showed a *T*_c_ from 94 K (i) for the YBCO substrate and 91 K (i), which is within the error of our measurement of the known *T*_c_ of ∼93. The zero-field-cooled (ZFC) measurement is shown in Figure 3d, which shows higher magnetic moments due to the lack of field lines at the time of superconducting transition.

As described above, compared to the YBCO substrate control (Figure 3, black lines), the general magnitude of repulsion diminishes with stronger magnetic fields, as shown where the magnetic moment is normalized by the mass of each sample (emu/g). This can also be observed in a critical current density calculation, which further adds the contribution of the sample holder dimensions (Figure S6), where A1–A3 show similar variance of *J*_c_, but at a lower magnitude and smaller *J*_c_ range when subjected to the substrate. The reduced *J*_c_ range suggests that A1–A3 may be more suitable for applications requiring consistent performance across varying magnetic field strengths, where a high maximum *J*_c_ is not critical. Due to the similarities of grain size and composition, we focused on the A sample series for magnetic measurements as well as the composite studies described next.

As described above, the YBCO colloids from the A- and T-series had good colloidal stability and could be suspended in solvents such as toluene. This allowed us to make concentrated inks or pastes that could be readily mixed with polymers or elastomers, such as poly­(dimethylsiloxane) (PDMS), and its variants, to form composites as illustrated in Figure 4a. Here, YBCO was first sonicated in a solvent and then added to the PDMS base at a desired weight percent (wt %), thoroughly mixed, and then the PDMS initiator was added. After further mixing, the gel was added to a mold, which could be either a preformed PDMS or any rigid negative shape. We found that in a typical PDMS experiment, special care was needed to purify the YBCO colloids free of excess ligands via additional centrifugation and a solvent/antisolvent washing steps.^62^ Excess ligands, especially OAm, prevented PDMS from fully curing. After being cured overnight, the PDMS molds were removed and tested.

**Figure 4:**
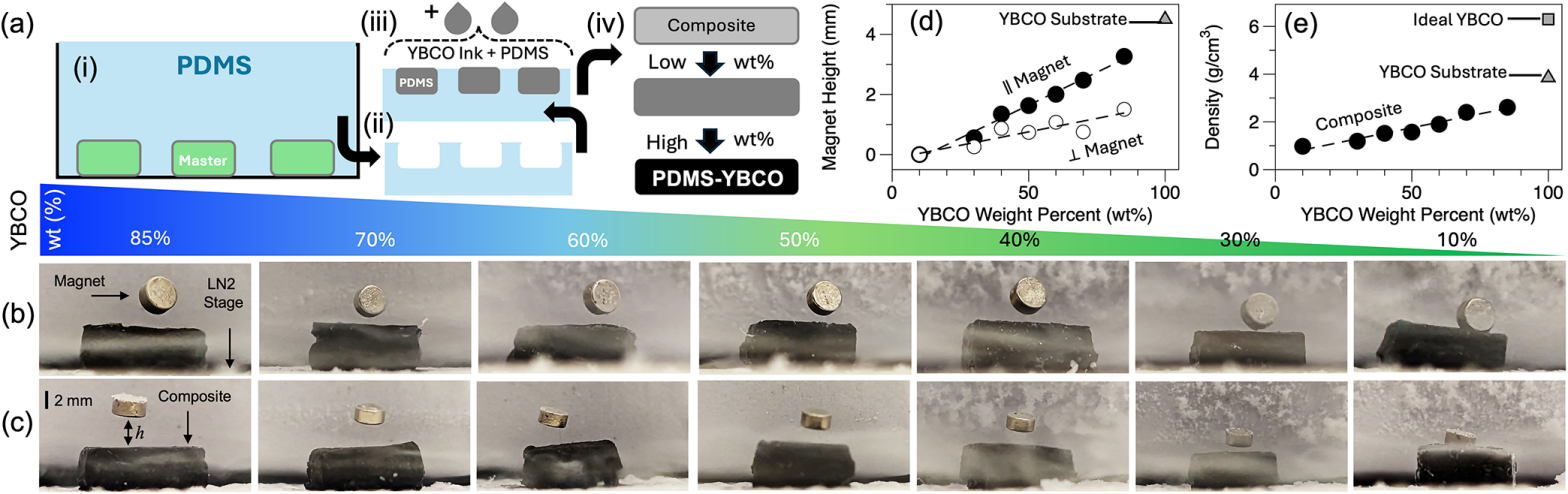
(a) Schematic of the PDMS negative mold preparation (i), PDMS mold used with master grids removed (ii), addition of YBCO + PDMS paste into the molds (iii), and removal of PDMS–YBCO elastomer composites (iv). Photographs of YBCO–PDMS composites with weight percentages shown, under LN_2_ cooling with an Nd magnet shown levitating using different parallel (b) and perpendicular (c) orientations. Measured Nd magnetic height from composite surface (d) and measured density (e) of each composite, along with the density of the YBCO pellet substrate. The literature reference YBCO solids are shown for comparison.

Figure 4b,c shows a series of photographs of YBCO+PDMS composite slabs at decreasing YBCO powder wt % from 85 to 10. When cooled in an LN_2_ bath, with an Nd magnetic placed on top, a Meissner effect is observed, and the Nd experiences strong quantum locking similar to that of the parent substrate shown in Figure 1a. The height (h) of the magnet provides a semiquantitative way to compare the composites and is plotted in Figure 4d. We found that h was sensitive to both magnet orientation (due to its change in flux lines), as well as composite sides, which was due to differences in local YBCO density (see below). However, a near-linear h-to-wt% trend was observed, showing the tunability of these composites without the need to adjust or engineer the material or construction. The composite densities were also measured, as plotted in Figure 4e, and showed an expected, but not perfect, linear trend. These density values highlight another important aspect of this work, as the prepared YBCO pellet, before being ground, had a density below that of its commercial or ideal value (see the point in Figure 4e), indicating that the starting material had some open space within the solid, which was also observed in the SEMs of Figure 1a, and may contribute to the ease of breaking down.

Figure 5 shows additional composites prepared by using a large-scale synthesis of A1–A3. Compared to the substrate YBCO at 70 wt % (a), and the same sample with additional 10% graphite (b), 70 wt % A1 (c) and 70 wt % A3 (d) had similar performance at 70 wt %, as shown qualitatively via similar heights of the quantum locked Nd magnet. Interestingly, all samples had a high degree of plasticity and flexibility that closely resembled that of PDMS, as shown in the photograph in Figure 5e. In addition to rectangular slabs or cubes, the PDMS could also take the form of any rigid mold, such as the patterned one shown in Figure 5f. The distribution of YBCO within the PDMS was not yet perfect, as shown in a low-resolution SEM cross section of the A1 PDMS–YBCO composite, shown in Figure 5g. Additional SEM and elemental analysis via EDS imaging are shown in Figure S7, which exhibits that YBCO concentration gradients increase toward the bottom of the composite. This indicates some settling during curing, which is the reason for differences in magnet heights in Figure 4, and can be easily overcome in future studies.

**Figure 5:**
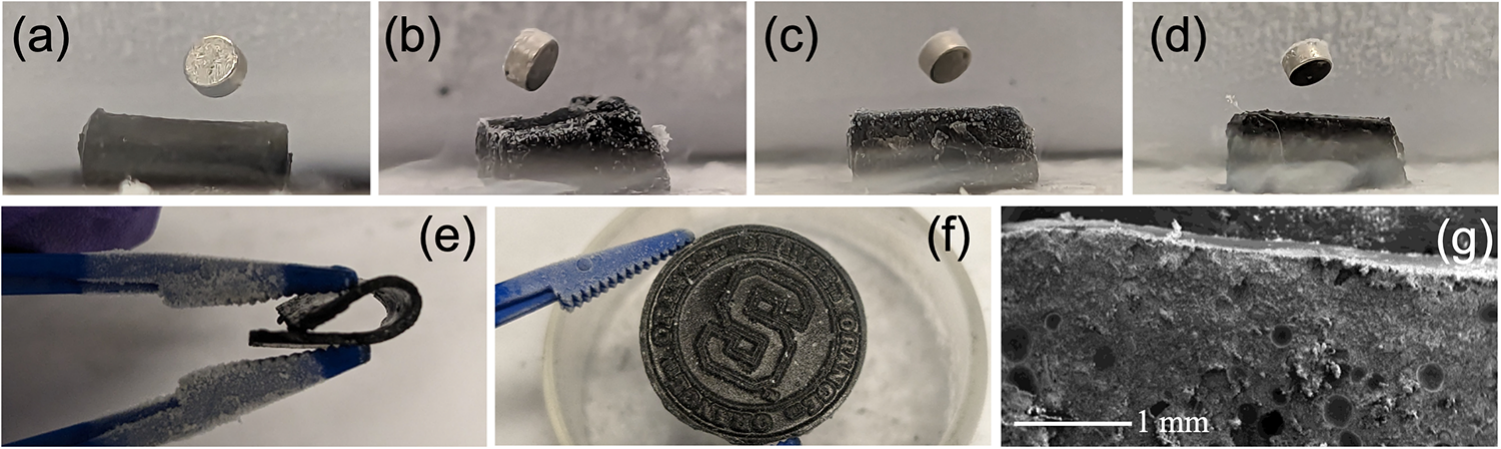
Photographs comparing YBCO–PDMS at 70 wt % (+10% graphite) (a), 70 wt % YBCO (b), 70 wt % A1 (c), and 70 wt % A3 (d) in LN_2_ with the Nd magnet. Photograph of examples of YBCO–PDMS flexibility (e) and moldability (f) while cooled to LN_2_ temperature. Low-resolution SEM image of the YBCO–PDMS cross section (g). See additional SEM and EDS in Figure S5.

The magnetic susceptibility of the YBCO–PDMS composites was also measured. Figure 6 shows measurements at 100 (a), 70 (b), and 5 K (c) for the 70 wt % YBCO–PDMS (orange) and 70 wt % plus 10 wt % graphite (80 wt % total, green) elastomer samples and compared to the YBCO substrate that has been normalized to 70% of the magnetic moment measured in Figure 3 (black). The results indicate that the final magnetic susceptibility of the composite closely matches that of YBCO itself, indicating that the PDMS does not alter the properties.

**Figure 6:**
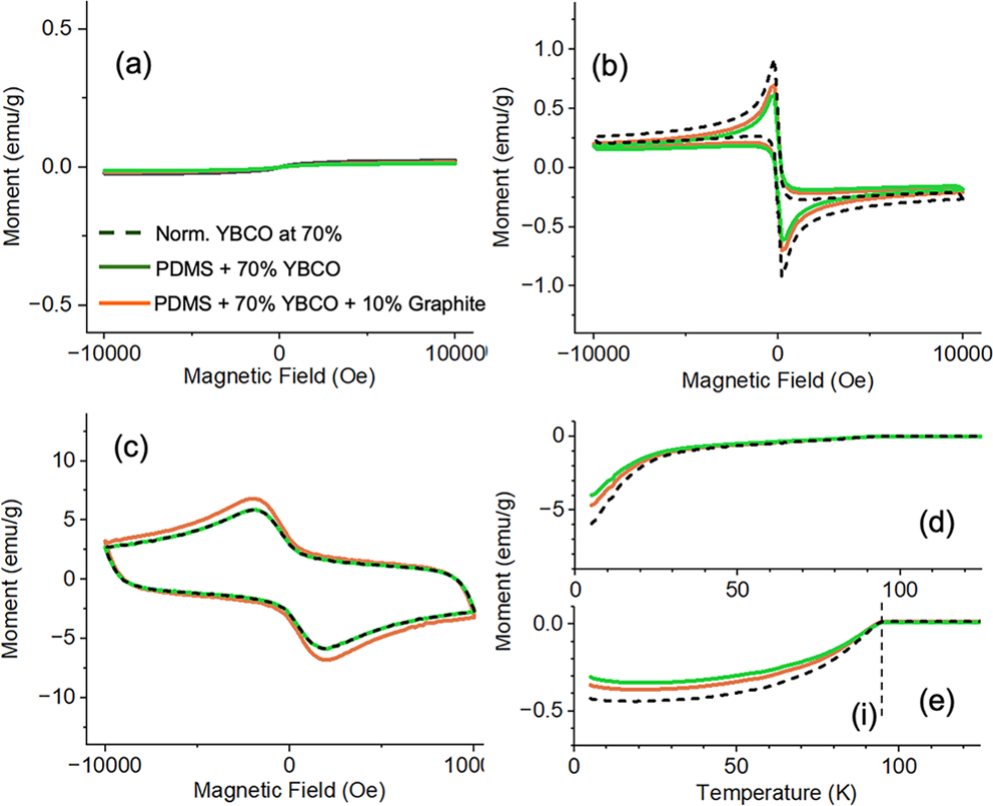
Magnetic susceptibility measurements at 100 (a), 70 (b), and 5 K (c) for the YBCO powder (normalized to 70% (black)), 70% YBCO–PDMS (green), and 70% YBCO–PDMS plus 10% graphite (orange). (d) Zero-field-cooled (ZFC) measurements. (e) Field-cooled (FC) measurements, with observed *T*_c_ (i) highlighted. Same color legend in each graph.

Taken together, these results show that it is possible to break down YBCO pellets or substrates into processable colloids without the loss of their crucial chemical stoichiometry or crystal structure. The use of ligands to chemically aid in the breakdown of YBCO grains also creates a hydrophobic surface that improves dispersity in solvents and elastomers. The ligand-to-YBCO molar ratios employed in this study provided the minimum needed to have favorable results, and higher excesses led to the dissolution of the solids and properties. It is clear that future work will focus on further fine-tuning of such ratios, exploring other ligand functionality and polarity, and the use of higher-powered sonication devices. Further, the demonstration here that the YBCO colloids can be readily dispersed in PDMS opens up many exciting avenues to create devices or functionality in elastomers. One interesting observation was that the elastomeric properties of the YBCO–PDMS composites were unaffected by the cryogenic LN_2_ cooling, and the composites have the same properties months after creation and after many freeze–thaw cycles. Another advantage this approach has is that it is likely transferable to other HTSC compositions and variations, and considering the multitude of types of elastomers, such as those with conductivity, it is clear that a wealth of conductivity and magnetoresistance measurements will advance this approach. Moreover, as shown in Figures 5 and S7, a third component, such a graphene or graphite, can readily disperse in the composite, further expanding properties and potential applications. Finally, as mentioned above, to date, it remains difficult to synthesize YBa_2_Cu_3_O_7−δ_ colloids or nanoparticles with uniform morphology and superconductive properties. It is possible that the top-down processing approach explored here may provide a method to create size-controlled colloids with a rational design advantage, which is part of our ongoing work.

## Conclusion

In this work, we explored the hypothesis that YBCO colloids could be created by the top-down chemical breakdown of solid substrates. This would allow for more processable YBCO with the crucial stoichiometry and superconducting properties of the parent material. We found that when combined with mechanical and ultrasonic treatment, ligands including oleyl amine, oleic acid, and trioctylphosphine and its oxide can create such colloids with a 1–3 μm grain size. X-ray diffraction confirmed crystal preservation during processing, and scanning electron microscopy confirmed substrate breakdown and colloidal morphologies. Critical superconducting properties of the YBCO were also preserved, as qualitatively shown via Meissner effects and quantum locking and quantitatively via magnetic susceptibility measurements. To test the processability of the colloids, inks or pastes were created in organic solvents, which were aided by the ligand-modified surfaces, and mixed with the elastomer PDMS. This allowed for the creation of elastomers with controlled weight percentages, whose soft composite mimicked the superconductivity of the YBCO parent. The elastomer composite was flexible and could be used to mold different shapes and features, which survived repeated cryogenic testing. The study opens a wealth of potential avenues to study as it relates to ligand type and concentration, other rare-earth YBCO analogues, sonication power, and elastomer or polymer types, properties, or additives, all of which can take advantage of the YBCO colloids for a range of applications.

## Supplementary Material



## Notes

Elemental analysis, photographs of A- and T-series samples, additional SEM and TEM micrographs, UV–vis, FTIR, and UV–vis results, critical current plots, and SEM of PDMS–YBCO composites (PDF)

## References

[1] Slimani Y., Hannachi E., Hamrita A., Ben Salem M. K., Ben Azzouz F., Manikandan A., Ben Salem M. (2018). Comparative
Investigation of the Ball Milling Role against Hand Grinding on Microstructure,
Transport and Pinning Properties of Y3Ba5Cu8O18±δ and YBa2Cu3O7-δ. Ceram. Int..

[2] Wei X., Peng E., Xie Y., Xue J., Wang J., Ding J. (2017). Extrusion Printing of a Designed
Three-Dimensional YBa _2_ Cu _3_ O _7–x_ Superconductor with Milled
Precursor Powder. J. Mater. Chem. C.

[3] Alami A. H., Assad M. A., Aokal C. (2016). Facile and
Cost-Effective Synthesis
and Deposition of a YBCO Superconductor on Copper Substrates by High-Energy
Ball Milling. Metall. Mater. Trans. A.

[4] Obradors X., Puig T. (2014). Coated Conductors for Power Applications: Materials Challenges. Supercond. Sci. Technol..

[5] Malozemoff A. P. (2012). Second-Generation
High-Temperature Superconductor Wires for the Electric Power Grid. Annu. Rev. Mater. Res..

[6] Frolov S. M., Manfra M. J., Sau J. D. (2020). Topological
Superconductivity in
Hybrid Devices. Nat. Phys..

[7] Bondarenko S. I., Koverya V. P., Krevsun A. V., Link S. I. (2017). High-Temperature
Superconductors of the Family (RE)­Ba2Cu3O7-δ and Their Application
(Review Article). Low Temp. Phys..

[8] Coombs T. A., Wang Q., Shah A., Hu J., Hao L., Patel I., Wei H., Wu Y., Coombs T., Wang W. (2024). High-Temperature Superconductors
and Their Large-Scale Applications. Nat. Rev.
Electr. Eng..

[9] Namburi D. K., Shi Y., Cardwell D. A. (2021). The processing and
properties of bulk (re)­bco high
temperature superconductors: current status and future perspectives. Supercond. Sci. Technol..

[10] Dadras S., Ghavamipour M. (2018). Properties of YBCO High Temperature Superconductor
Synthesized by Microwave Oven. Mater. Res. Express.

[11] Gandhi A. C., Lin J. G. (2018). Microwave-Assisted
Synthesis and Critical Analysis
for YBa _2_ Cu _3_ O _6+*δ*
_ Nanoparticles. Supercond. Sci. Technol..

[12] Jasim S. E., Jusoh M. A., Hafiz M., Jose R. (2016). Fabrication of Superconducting
YBCO Nanoparticles by Electrospinning. Procedia
Eng..

[13] Obradors X., Puig T., Pomar A., Sandiumenge F., Mestres N., Coll M., Cavallaro A., Romà N., Gázquez J., González J. C., Castaño O., Gutierrez J., Palau A., Zalamova K., Morlens S., Hassini A., Gibert M., Ricart S., Moretó J. M., Piñol S., Isfort D., Bock J. (2006). Progress towards
All-Chemical Superconducting YBa _2_ Cu _3_ O _7_ -Coated Conductors. Supercond. Sci.
Technol..

[14] Pavan
Kumar Naik S., Missak Swarup Raju P. (2016). 1 School of Physics, University of
Hyderabad, Hyderabad-500046, India. Microstructural and Magnetic Properties
of YBCO Nanorods: Synthesized by Template Growth Method. AIMS Mater. Sci..

[15] Thuy T. T., Hoste S., Herman G. G., De Buysser K., Lommens P., Feys J., Vandeput D., Van Driessche I. (2009). Sol–Gel
Chemistry of an Aqueous Precursor Solution for YBCO Thin Films. J. Sol–Gel Sci. Technol..

[16] Li F., Vipulanandan C. (2003). Production
and Characterization of YBCO Nanoparticles. IEEE Trans. Appl. Supercond..

[17] Zhu Z., Gao D., Dong C., Yang G., Zhang J., Zhang J., Shi Z., Gao H., Luo H., Xue D. (2012). Coexistence of Ferromagnetism
and Superconductivity in YBCO Nanoparticles. Phys. Chem. Chem. Phys..

[18] Xu X. L., Guo J. D., Wang Y. Z., Sozzi A. (2002). Synthesis of Nanoscale
Superconducting YBCO by a Novel Technique. Physica
C.

[19] Vase P., Yueqiang S., Freltoft T. (1990). Deposition, Characterization,
and
Laser Ablation Patterning of YBCO Thin Films. Appl. Surf. Sci..

[20] Opherden L., Sieger M., Pahlke P., Hühne R., Schultz L., Meledin A., Van Tendeloo G., Nast R., Holzapfel B., Bianchetti M., MacManus-Driscoll J. L., Hänisch J. (2016). Large Pinning Forces and Matching
Effects in YBa2Cu3O7-δ Thin Films with Ba2Y­(Nb/Ta)­O6 Nano-Precipitates. Sci. Rep..

[21] Kamba, S. ; Petzelt, J. ; Zelenzy, V. ; Pechen, E. V. ; Krasnosvobodtsev, S. I. ; Gorshunov, B. P. Infrared reflectance of anisotropic (II0) YBCO epitaxial film Solid State Commun. 1989, 70 5 547–551 10.1016/0038-1098(89)90947-2.

[22] Jang E.-S., Chang J.-J., Jeon S.-H., Khim Z.-G., Choy J.-H. (2005). Electrophoretic
Route to Bi_2_ Sr_2_ CaCu_2_ O_8+*y*
_ Films and Microfibers from Superconducting Colloids. Adv. Mater..

[23] Hannachi E., Slimani Y., Ben Salem M. K., Hamrita A., Mani D. K., Ben Salem M., Ben Azzouz F. (2015). Magneto-Conductivity Fluctuation
in YBCO Prepared by Sintering of Ball-Milled Precursor Powder. Mater. Chem. Phys..

[24] Hamrita A., Ben Azzouz F., Madani A., Ben Salem M. (2012). Magnetoresistivity
and Microstructure of YBa2Cu3Oy Prepared Using Planetary Ball Milling. Physica C.

[25] Hannachi E., Ben Salem M. K., Slimani Y., Hamrita A., Zouaoui M., Ben Azzouz F., Ben Salem M. (2013). Dissipation
Mechanisms in Polycrystalline
YBCO Prepared by Sintering of Ball-Milled Precursor Powder. Physica B.

[26] Tong Y., Bladt E., Aygüler M. F., Manzi A., Milowska K. Z., Hintermayr V. A., Docampo P., Bals S., Urban A. S., Polavarapu L., Feldmann J. (2016). Highly Luminescent Cesium Lead Halide
Perovskite Nanocrystals with Tunable Composition and Thickness by
Ultrasonication. Angew. Chem., Int. Ed..

[27] Okejiri F., Zhang Z., Liu J., Liu M., Yang S., Dai S. (2020). Room-Temperature Synthesis of High-Entropy Perovskite Oxide Nanoparticle
Catalysts through Ultrasonication-Based Method. ChemSusChem.

[28] Nguyen V. S., Rouxel D., Hadji R., Vincent B., Fort Y. (2011). Effect of
Ultrasonication and Dispersion Stability on the Cluster Size of Alumina
Nanoscale Particles in Aqueous Solutions. Ultrason.
Sonochem..

[29] Prabhakaran D., Subramanian C., Balakumar S., Ramasamy P. (1999). Morphology and Etching
Studies on YBCO and CuO Single Crystals. Physica
C.

[30] Villarejo B., Pino F., Pop C., Ricart S., Vallès F., Mundet B., Palau A., Roura-Grabulosa P., Farjas J., Chamorro N., Yáñez R., Granados X., Puig T., Obradors X. (2021). High Performance of
Superconducting YBa_2_ Cu_3_ O_7_ Thick
Films Prepared by Single-Deposition Inkjet Printing. ACS Appl. Electron. Mater..

[31] Roma N., Morlens S., Ricart S., Zalamova K., Moreto J. M., Pomar A., Puig T., Obradors X. (2006). Acid Anhydrides: A
Simple Route to Highly Pure Organometallic Solutions for Superconducting
Films. Supercond. Sci. Technol..

[32] Coll M., Ye S., Rouco V., Palau A., Guzman R., Gazquez J., Arbiol J., Suo H., Puig T., Obradors X. (2013). Solution-Derived
YBa_2_ Cu_3_ O_7_ Nanocomposite Films with
a Ba_2_ YTaO_6_ Secondary Phase for Improved Superconducting
Properties. Supercond. Sci. Technol..

[33] Rijckaert H., Malmivirta M., Banerjee S., Billinge S. J. L., Huhtinen H., Paturi P., De Buysser K., Van Driessche I. (2022). Superconducting
HfO_2_ -Added Solution-Derived YBa_2_ Cu_3_ O_7_ Nanocomposite Films: The Effect of Colloidal Nanocrystal
Shape and Crystallinity on Pinning Mechanism. Supercond. Sci. Technol..

[34] De
Keukeleere K., Cayado P., Meledin A., Vallès F., De Roo J., Rijckaert H., Pollefeyt G., Bruneel E., Palau A., Coll M., Ricart S., Van Tendeloo G., Puig T., Obradors X., Van Driessche I. (2016). Superconducting
YBa_2_ Cu_3_ O_7−δ_ Nanocomposites
Using Preformed ZrO_2_ Nanocrystals: Growth Mechanisms and
Vortex Pinning Properties. Adv. Electron. Mater..

[35] Rijckaert H., Pollefeyt G., Sieger M., Hänisch J., Bennewitz J., De Keukeleere K., De Roo J., Hühne R., Bäcker M., Paturi P., Huhtinen H., Hemgesberg M., Van Driessche I. (2017). Optimizing Nanocomposites through Nanocrystal Surface
Chemistry: Superconducting YBa_2_ Cu_3_ O_7_ Thin Films via Low-Fluorine Metal Organic Deposition and Preformed
Metal Oxide Nanocrystals. Chem. Mater..

[36] Li Z., Coll M., Mundet B., Chamorro N., Vallès F., Palau A., Gazquez J., Ricart S., Puig T., Obradors X. (2019). Control of Nanostructure and Pinning Properties in
Solution Deposited YBa2Cu3O7–x Nanocomposites with Preformed
Perovskite Nanoparticles. Sci. Rep..

[37] Obradors X., Puig T., Ricart S., Coll M., Gazquez J., Palau A., Granados X. (2012). Growth, Nanostructure
and Vortex
Pinning in Superconducting YBa_2_ Cu_3_ O_7_ Thin Films Based on Trifluoroacetate Solutions. Supercond. Sci. Technol..

[38] Rijckaert H., De Roo J., Roeleveld K., Pollefeyt G., Bennewitz J., Bäcker M., Lynen F., De Keukeleere K., Van Driessche I. (2017). Microwave-assisted
YBa_2_ Cu_3_ O_7_ Precursors: A Fast and
Reliable Method towards Chemical Precursors
for Superconducting Films. J. Am. Ceram. Soc..

[39] Saltarelli L., Gupta K., Rasi S., Kethamkuzhi A., Queraltó A., Garcia D., Gutierrez J., Farjas J., Roura-Grabulosa P., Ricart S., Obradors X., Puig T. (2022). Chemical and Microstructural
Nanoscale Homogeneity in Superconducting
YBa_2_ Cu_3_ O _7– *x*
_ Films Derived from Metal-Propionate Fluorine-Free Solutions. ACS Appl. Mater. Interfaces.

[40] Saltarelli L., Sanchez-Rodriguez D., Gupta K., Kethamkuzhi A., Farjas J., Molins E., Yañez R., Ricart S., Obradors X., Puig T. (2024). Metal Propionate
Solutions
for High-Throughput Liquid-Assisted Manufacturing of Superconducting
REBa_2_ Cu_3_ O_7‑δ_ (RE =
Y, Gd, Sm, and Yb) Films. ACS Appl. Mater. Interfaces.

[41] Thedford R. P., Yu F., Tait W. R. T., Shastri K., Monticone F., Wiesner U. (2023). The Promise of Soft-Matter-Enabled Quantum Materials. Adv. Mater..

[42] Larbalestier D., Gurevich A., Feldmann D. M., Polyanskii A. (2001). High-Tc Superconducting
Materials for Electric Power Applications. Nature.

[43] Shani L., Michelson A. N., Minevich B., Fleger Y., Stern M., Shaulov A., Yeshurun Y., Gang O. (2020). DNA-Assembled Superconducting
3D Nanoscale Architectures. Nat. Commun..

[44] Zolotavin P., Guyot-Sionnest P. (2010). Meissner Effect in Colloidal Pb Nanoparticles. ACS Nano.

[45] She J.-L., Liu R.-S. (2008). A Simplified Synthetic
Experiment of YBa2Cu3O7–x
Superconductor for First-Year Chemistry Laboratory. J. Chem. Educ..

[46] Hull J. R., Murakami M. (2004). Applications of Bulk High-Temperature Superconductors. Proc. IEEE.

[47] Holland, H. J. ; Murtagh, M. J. An XRD Morphology Index for Talcs: The Effect of Particle Size and Morphology on the Specific Surface Area. In Advances in X-ray Analysis; ICDD, 2000; Vol. 42.

[48] Lu C.-H., Yeh C.-H. (2000). In̅uence
of Hydrothermal Conditions on the Morphology
and Particle Size of Zinc Oxide Powder. Ceram.
Int..

[49] Mclaren A., Valdes-Solis T., Li G., Tsang S. C. (2009). Shape and Size Effects
of ZnO Nanocrystals on Photocatalytic Activity. J. Am. Chem. Soc..

[50] Zhang L., Belova V., Wang H., Dong W., Möhwald H. (2014). Controlled
Cavitation at Nano/Microparticle Surfaces. Chem.
Mater..

[51] Mason T. J. (1997). Ultrasound
in Synthetic Organic Chemistry. Chem. Soc. Rev..

[52] Peng S., Wang C., Xie J., Sun S. (2006). Synthesis
and Stabilization
of Monodisperse Fe Nanoparticles. J. Am. Chem.
Soc..

[53] Li J. J., Wang Y. A., Guo W., Keay J. C., Mishima T. D., Johnson M. B., Peng X. (2003). Large-Scale Synthesis of Nearly Monodisperse
CdSe/CdS Core/Shell Nanocrystals Using Air-Stable Reagents via Successive
Ion Layer Adsorption and Reaction. J. Am. Chem.
Soc..

[54] Wang F., Tang R., Buhro W. E. (2008). The Trouble with TOPO; Identification
of Adventitious Impurities Beneficial to the Growth of Cadmium Selenide
Quantum Dots, Rods, and Wires. Nano Lett..

[55] Jasper J.
J. (1972). The Surface
Tension of Pure Liquid Compounds. J. Phys. Chem.
Ref. Data.

[56] Chumpitaz L. D. A., Coutinho L. F., Meirelles A. J. A. (1999). Surface
Tension of Fatty Acids and
Triglycerides. J. Am. Oil Chem. Soc..

[57] Dang D. K., Kim E. J. (2015). Solvothermal-Assisted Liquid-Phase Exfoliation of Graphite
in a Mixed Solvent of Toluene and Oleylamine. Nanoscale Res. Lett..

[58] Choudhury, A. K. R. Repellent Finishes. In Principles of Textile Finishing; Elsevier, 2017; pp 149–194.

[59] Plesset M. S., Prosperetti A. (1977). Bubble Dynamics and Cavitation. Annu. Rev. Fluid Mech..

[60] Mbewana-Ntshanka N. G., Moloto M. J., Mubiayi P. K. (2020). Role of
the Amine and Phosphine Groups
in Oleylamine and Trioctylphosphine in the Synthesis of Copper Chalcogenide
Nanoparticles. Heliyon.

[61] Shipra, Gomathi A., Sundaresan A., Rao C. N. R. (2007). Room-Temperature Ferromagnetism in Nanoparticles of
Superconducting Materials. Solid State Commun..

[62] Yang Y., Li J., Lin L., Peng X. (2015). An Efficient and Surface-Benign Purification
Scheme for Colloidal Nanocrystals Based on Quantitative Assessment. Nano Res..

