# Supplementary material for: Preparing Superconducting YBCO Colloids via a Top-Down Processing Route for Applications in Soft Composites
Source: ACS Omega. 2025 Nov 12;10(46):56401–10. doi: 10.1021/acsomega.5c08369 (PMC12658815; doi:10.1021/acsomega.5c08369)
Supplement: Supplementary file 1 [file ao5c08369_si_001.pdf]

Preparing Superconducting YBCO Colloids via a Top Down Processing Route for Applications in Soft Composites

Harrison Reinheimer, Mathew M. Maye*

Department of Chemistry, Syracuse University, Syracuse New York 13244 U.S.A.
**mmmaye@syr.edu*

SUPPORTING INFORMATION

Table S1: Molar ratios determined via elemental analysis (ICP-OES)

	Molar Ratio		
Sample	[Y]	[Ba]	[Cu]
**S**	1	1.85	3.85

S = YBCO substrate powders

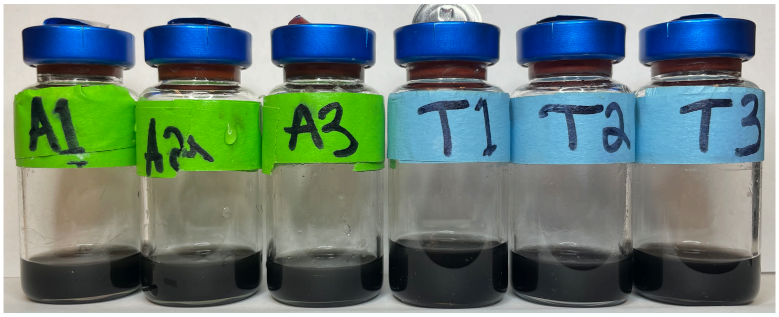

Figure S1 : Photographs of YBCO samples suspended in solvent with labels shown.

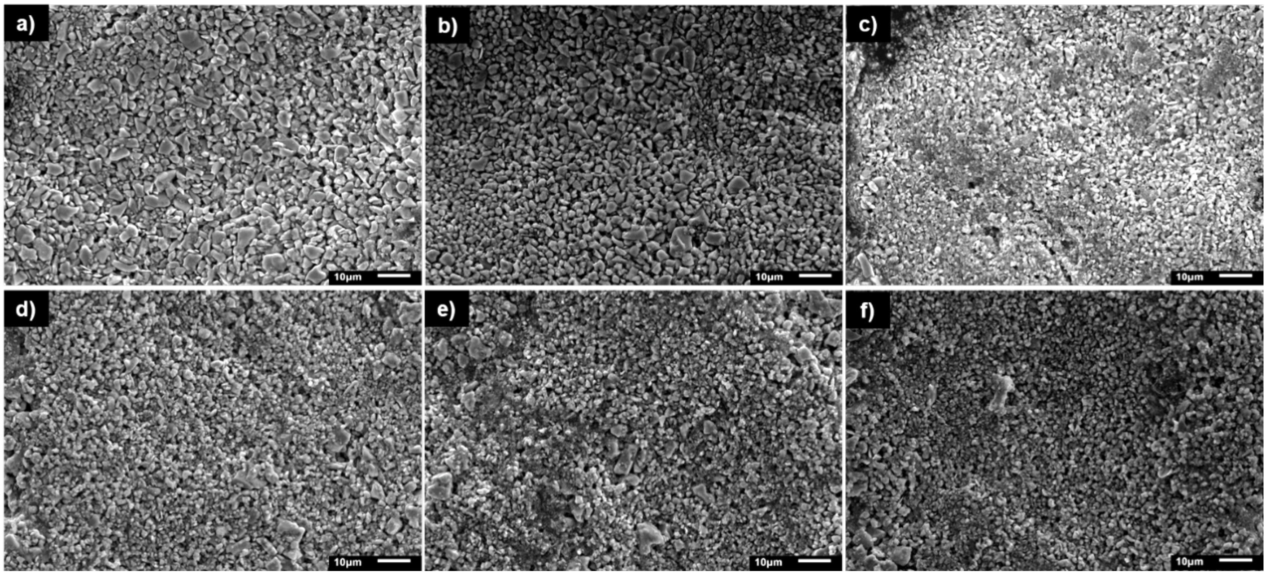

Figure S2 : SEM micrographs of YBCO colloids in samples A1 (a), A2 (b), A3 (c), T1 (d), T2 (e) and T3 (f) at 100x.

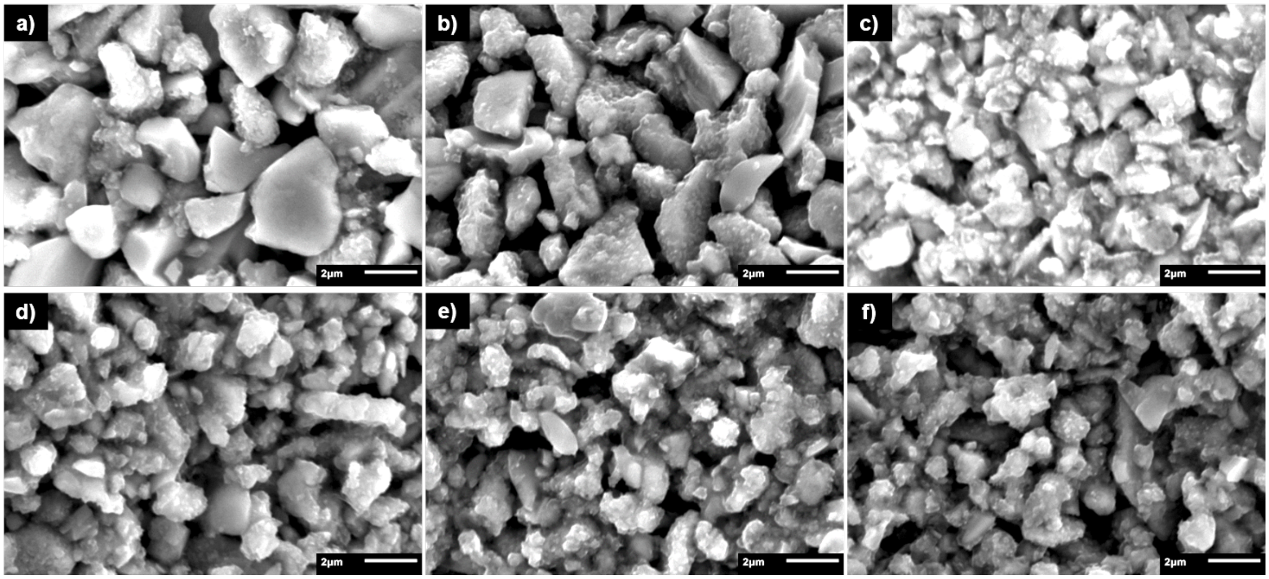

Figure S3 : SEM micrographs of YBCO colloids in samples A1 (a), A2 (b), A3 (c), T1 (d), T2 (e) and T3 (f) at 3000x.

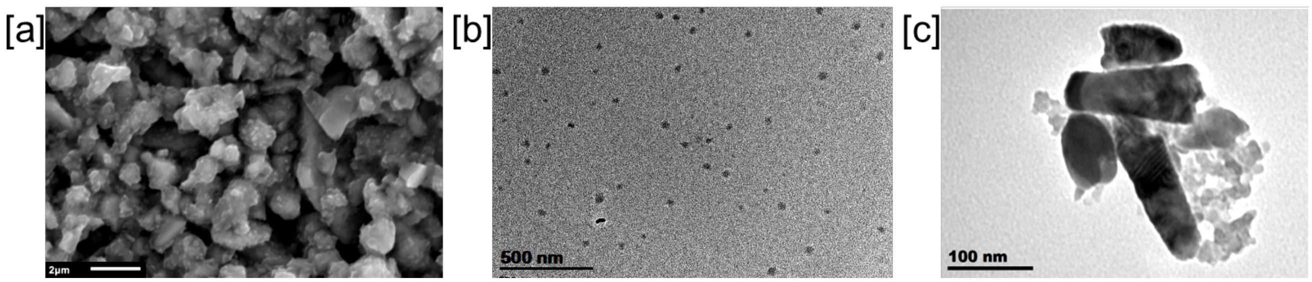

Figure S4: Comparison of T3 SEM (a), T3 TEM at low magnification (b), and T3 TEM at high magnification (c) revealing nanoscale YBCO colloids.

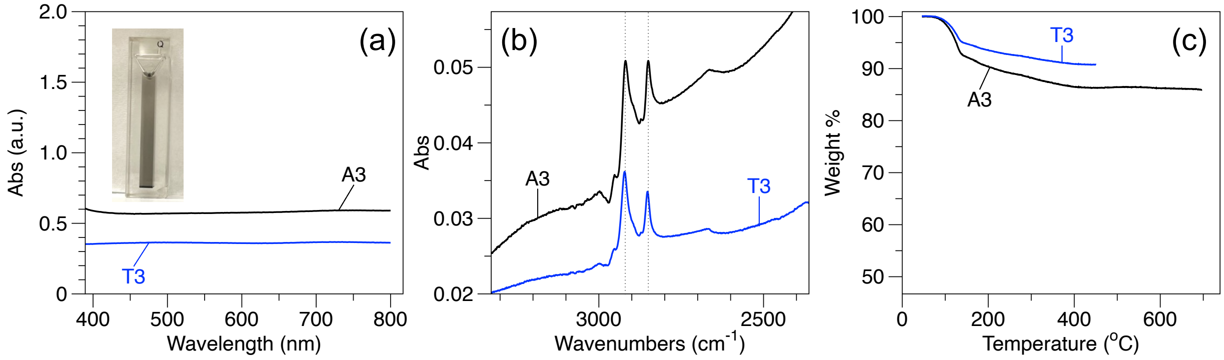

Figure S5: Representative UV-vis (a), FTIR (b), and TGA (c) characterization of purified A3 and T3 samples. UV-vis samples were redispersed in toluene, while FTIR and TGA used dried powders. Inset: Photograph of cuvette holding A3 sample.

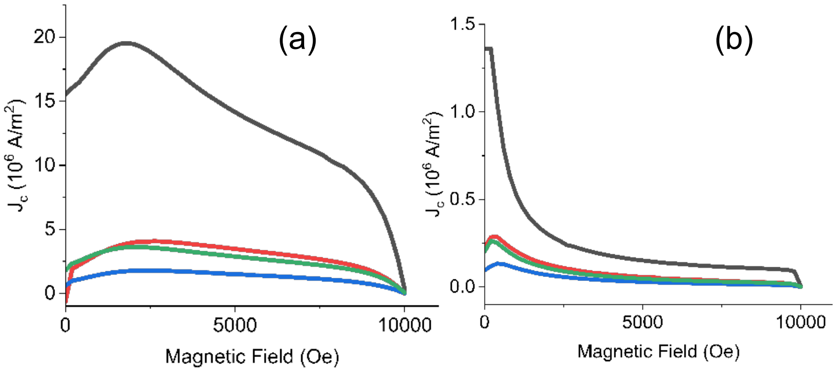

$$J_C = \frac{3\Delta M}{2r}$$

Figure S6: Critical current density (Jc) calculations for YBCO substrate powder (black), and samples A1(red), A2(blue), A3(green) at (a) 5 K and (b) 70 K. The Jc values were calculated according to Jc = 3ΔM/2*r* where ΔM is the difference in magnetization (M) and *r* is radius of the cylindrical sample holder.

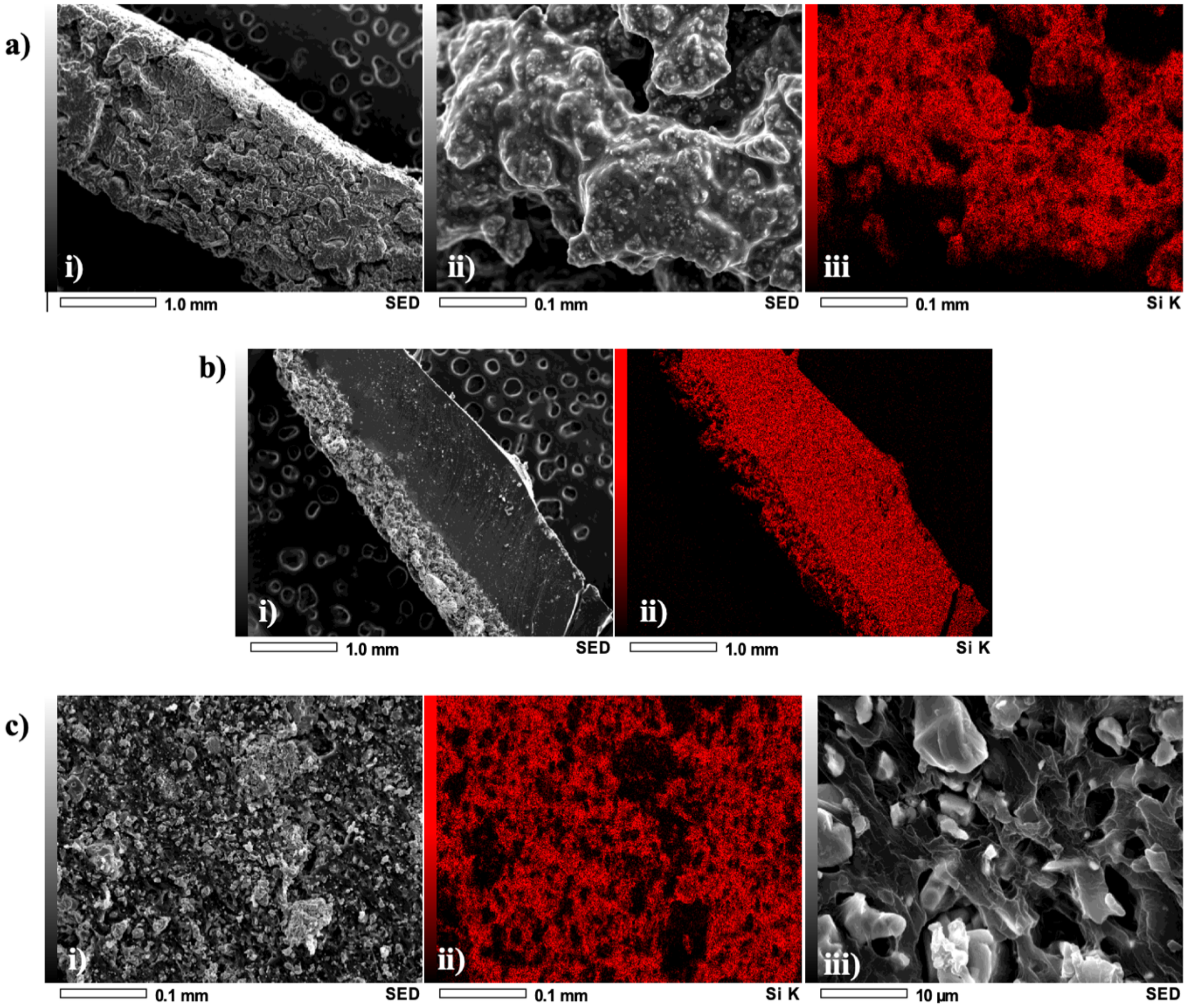

Figure S7: (a) A collection of SEM micrographs for the A3 70 wt% PDMS-YBCO composite at 30x (i), 300x (ii), and the Si EDS scan at 300x(iii). (b) SEM of a A3 30% wt% PDMS-YBCO composite mple/PDMS. 30x(i), 30x Si EDS(ii). (c) A1, 70% composite Sample/PDMS. 300x(i), Si EDS at 300x(ii), 3000x(iii).
